# Expression and Roles of Antimicrobial Peptides in Innate Defense of Airway Mucosa: Potential Implication in Cystic Fibrosis

**DOI:** 10.3389/fimmu.2020.01198

**Published:** 2020-06-30

**Authors:** Regina Geitani, Carole Ayoub Moubareck, Zhengzhong Xu, Dolla Karam Sarkis, Lhousseine Touqui

**Affiliations:** ^1^Microbiology Laboratory, School of Pharmacy, Saint Joseph University, Beirut, Lebanon; ^2^College of Natural and Health Sciences, Zayed University, Dubai, United Arab Emirates; ^3^Jiangsu Key Laboratory of Zoonosis, Yangzhou University, Yangzhou, China; ^4^Sorbonne Université, INSERM UMR_S 938, Centre de Recherche Saint Antoine (CRSA), Paris, France; ^5^“Mucoviscidose and Bronchopathies Chroniques”, Pasteur Institute, Paris, France

**Keywords:** respiratory infections, antibiotic resistance, antimicrobial peptides, antimicrobial effect, immune modulation, cystic fibrosis

## Abstract

The treatment of respiratory infections is associated with the dissemination of antibiotic resistance in the community and clinical settings. Development of new antibiotics is notoriously costly and slow; therefore, alternative strategies are needed. Antimicrobial peptides (AMPs), the central effector molecules of the immune system, are being considered as alternatives to conventional antibiotics. Most AMPs are epithelium-derived and play a key role in host defense at mucosal surfaces. They are classified on the basis of their structure and amino acid motifs. These peptides display a range of activities, including not only direct antimicrobial activity, but also immunomodulation and wound repair. In the lung, airway epithelial cells and neutrophils, in particular, contribute to AMP synthesis. The relevance of AMPs for host defense against infection has been demonstrated in animal models and is supported by observations in patient studies, showing altered expression and/or unfavorable circumstances for their action in a variety of lung diseases. Of note, AMPs are active against bacterial strains that are resistant to conventional antibiotics, including multidrug-resistant bacteria. Several strategies have been proposed to use these peptides in the treatment of infections, including direct administration of AMPs. In this review, we focus on studies related to direct bactericidal effects of AMPs and their potential clinical applications with a particular focus on cystic fibrosis.

## Historical Overview and Definition

In the early 1920s, Fleming independently discovered both AMPs and penicillin. In 1922, he identified, in his nasal discharge, an antimicrobial substance, later named lysozyme, which was able to kill certain bacteria in few minutes. Seven years later, penicillin was carried forward for clinical application (1). After that, several AMPs were isolated and identified as having activity against both Gram-positive and Gram-negative bacteria. In 1939, gramicidin was the first natural peptide-based drug to be introduced in the market. It was isolated from *Bacillus brevis* and was active against a wide range of Gram-positive and some Gram-negative bacteria but was not devoid of toxicity (2). The real explosion of therapeutic potential of AMPs began in the early 1980s when Hans Boman isolated and characterized AMPs, known as cecropins, from the hemolymph of silk moth (*Hyalophora cecropia*) (3). Later in 1987, the significance of AMPs was increased when Zasloff discovered magainins in frog skin (*Xenopus laevis*) (4) and showed for the first time that AMPs are present not only in lower invertebrates but also in higher vertebrates (5). Antimicrobial activities in fluids such as blood, saliva, plasma, sweat, leucocytes secretions, and granule extracts were discovered at that period, suggesting the natural production of AMPs in humans (6). Since then, more than 3,000 naturally occurring AMPs have been isolated from different kingdoms (bacteria, archea, protists, fungi, plants, animals, and humans) and were registered in the AMP database (http://aps.unmc.edu/AP/main.php). Thus, AMPs were discovered at the same time as antibiotics (ATBs) but were eclipsed by the success of those drugs. Now that the emergence of ATB resistance is a major threat to human health, global voices are calling for solutions. Among the existing research lines for alternatives to conventional ATBs, AMPs, both natural and synthetic, seem to be promising candidates (7).

AMPs, also referred to as host defense peptides, are biologically active molecules with a rapid and broad spectrum of activity against bacteria, yeast, viruses, and fungi in addition to immunomodulatory activities, wound healing, and cytotoxic effects on cancer cells (8, 9). To date, the large majority of identified AMPs are antibacterial peptides representing 83% of all AMPs (10). AMPs, evolutionarily conserved in the genome, are produced by most living organisms as an essential component of their innate immune system, representing an ancient host defense mechanism to eliminate invading pathogens and boost immune response. In mammals, the primary site at which a host encounters a pathogen is classically the skin or the mucosal surface, such as the respiratory tract, the gastrointestinal tract, and the urogenital tract (11). Infections at these sites are prevented by the innate host defense responses intended to maintain host integrity (12). AMPs, being an important component of the innate immune system, constitute one of the early, rapid, nonspecific mechanisms by which the host immune system provides protection against infections (13). Studies using knockout mice and transgenic (Tg) expression systems have confirmed that AMPs play a major role in limiting microbial proliferation to skin and mucosal surfaces, therefore preventing spread to the deep tissues where serious infection may occur (14). AMPs are produced by epithelial cells of vertebrates as a first line of defense against microbial pathogens.

## Amps: Structure and Classification

**Figure 1:**
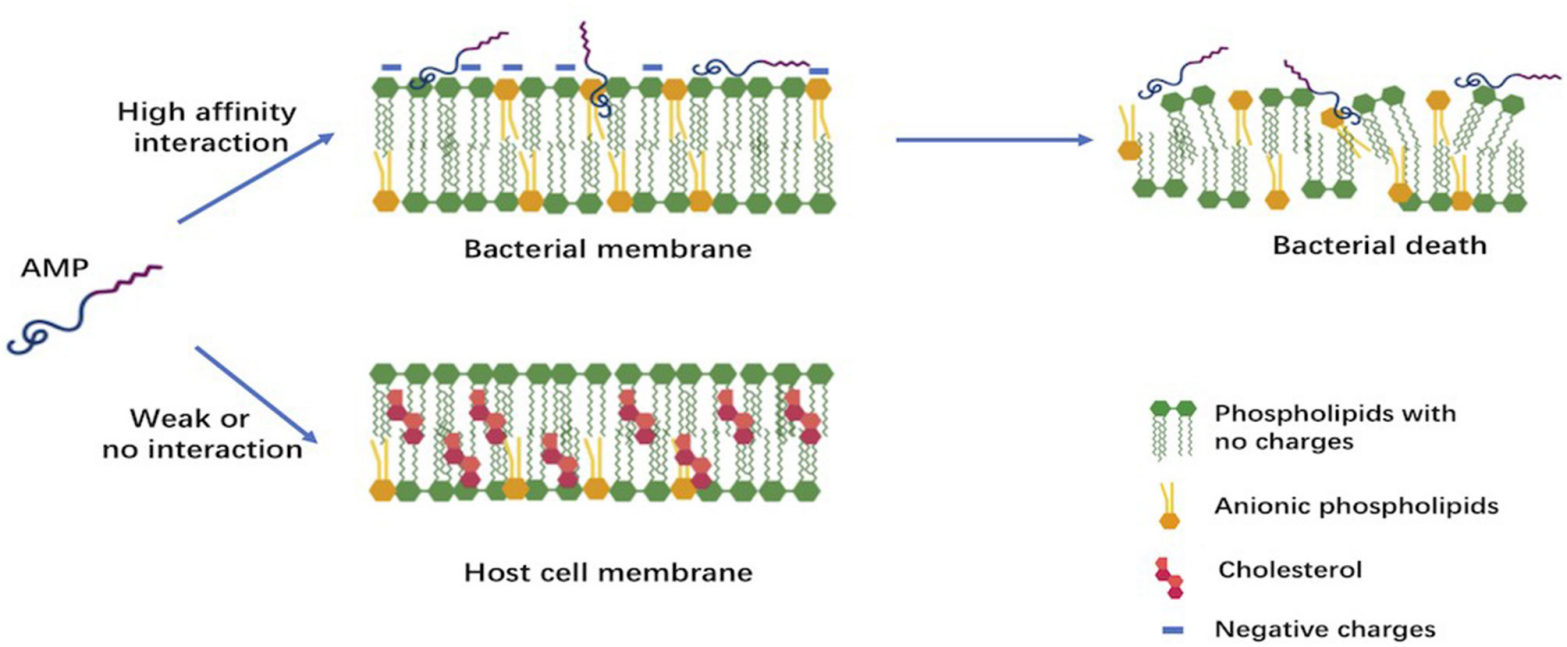
Early interactions of cationic antimicrobial peptides with bacterial or host cell membrane. The anionic molecules in the membranes of Gram-negative and Gram-positive bacteria attract cationic AMPs via electrostatic and hydrophobic interactions. In contrast to bacteria, the cytoplasmic membrane of host cells with a neutral net charge connects with cationic AMPs via hydrophobic interactions, which are relatively weak.

Despite their extreme diversity in terms of composition and length, AMPs share several common structural characteristics (15). The most studied AMPs are short polypeptides of fewer than 50 amino acids, cationic with an average net charge of +3, and having a hydrophobic content of 42% on average. Both the net positive charge and the hydrophobicity of these AMPs generate the observed amphipathic structure. This structure determines their conformational flexibility, enables electrostatic attraction between these cationic peptides and the anionic bacterial membranes, and allows penetration into bacterial cells inducing membrane lysis. Cationic AMPs, however, do not affect the neutrally charged mammalian cells; this chemical property favors their use as future drugs (7, 15). The differences in composition between bacterial cell membranes rich in phosphatidylglycerols and human cell membranes dominated by zwitterionic phospholipids is believed to be the major reason of the selectivity of AMPs (Figure 1) (7).

Based on structural features, AMPs can be classified into three subgroups: α-helical, β-sheet, and extended AMPs (16–18). These structures are highly correlated with the functional specificity of each peptide. Some of these peptides demonstrate no secondary structure in aqueous solution but become structured when exposed to a lipid, such as the bacterial cell membrane (19). In addition to that, some peptides might have mixed α-helical and β-sheet structures; classification is then based on the predominant one (20).

The first subgroup contains AMPs that form α-helical structures and are predominately found in the extracellular matrix of frogs and insects in addition to the extensively studied human AMP LL-37, which is a member of the cathelicidins. Cathelicidins, originally isolated from granule extracts of bovine neutrophils (21), are among the most diverse AMPs of vertebrates; they can adopt a variety of structures and play, in addition to their antimicrobial activity, an important immunomodulatory role (22). Magainins, which are active against a broad spectrum of microbial agents, present another example of AMPs with an α-helical structure. They have been extensively studied and are among the first ones to have been tested clinically (23). Cecropin is a prototype of this group and is active against Gram-negative bacteria. Other cecropins, which can act synergistically against both Gram-negative and Gram-positive bacteria, have been recently identified (24). Another final example of the α-helical AMPs is the aureins that are secreted from the granular dorsal glands of the Australian Green and Golden Bell Frog *Litoria aurea* and the southern Bell Frog *L. raniformis*. The aurein family is mostly active against Gram-positive bacteria, such as *Staphylococcus aureus* and *S. epidermidis*, and have anti-cancer activities (20).

The second subgroup includes cyclic AMPs that adopt a β-sheet structure, such as protegrins, defensins, and tachyplesins. Although they have antifungal properties in some cases, they are often considered to be antibacterial peptides (19). Defensins, the largest group of AMPs produced by mammals, were first discovered in human neutrophils as small cationic molecules. They have been found later in mammals, insects, plants, parasites, and fungi. Defensins are also involved in immune and inflammation responses (25). Although most defensins lose much of their antimicrobial activity at the physiological concentrations of Na^+^, Mg^2+^, or Ca^2+^, they have been shown to exhibit broad-spectrum antimicrobial activity against bacteria, fungi, and enveloped viruses *in vitro*. Of note, electrolytes may have a more complex effect on peptide-induced antimicrobial effects (25). Another example of β-sheet AMPs are tachyplesins, isolated from hemocytes of horseshoe crabs (20).

The third and last subgroup comprises peptides with a unique extended/random coil structure. In this category, most of the AMPs are from the cathelicidin family, which are known to have linear structure rather than secondary structure due to the presence of proline residues. One of the best studied peptides in this subgroup is indolicidin, which is produced by bovine leucocytes and consists of only 13 amino acids (17, 20).

Sources of some AMPs, their classes, and chemical structures are shown in Table 1.

**Table 1:** Classification of some antimicrobial peptides along with their chemical structure and origin.

**Classification**	**AMP**	**Origin**	**Chemical structure**
α-helix	LL-37 Melittin Dermaseptin-S1	Human Honey bee Frog	GIGAVLKVLTTGLPALISWIKRKRQQ GIGKFLHSAGKFGKAFVGEIMKS LLGDFFRKSKEIGEFKRIVQRIKDFLRNLVPRTES
β-helix	Protegrin-1 HNP-1 HBD-1	Pig Human Human	RGGRLC[1]YC[2]RRRFC[2]VC[1]VGR AC[1]YC[2]RIPAC[3]IAGGRRYGTC[2]IYGGRKWAFC[3]C[1] DHYNC[1]VSSGGQC[2]LYASC[3]PIFTKIQGTC[2]YRGKAKC[1]C[3]K
Extended structure	PR-39 Indolicidin Tritrpticin	Pig Cow Pig	RRRPRPPYLPRRPRPPFFPPLRLPPRIPPGFPPRFPPRPFP ILPWKWPWWPWRR VRRFPWWWPFLRR

*AMP, antimicrobial peptide; HBD-1, human β-defensin 1; HNP, human neutrophil defensing*.

## Mode of Action of AMPS

Enhanced understanding of the mechanism of action (MOA) of AMPs is of great importance to facilitate further development of peptide-based drugs as therapeutic agents. The MOA can be divided into two major classes: direct antimicrobial activity and immune modulation (16). Although it has been thought for many years that membrane destabilization was the sole direct MOA of AMPs against bacteria, additional mechanisms have been described. These MOA embrace non-membrane targeting mechanisms, including inhibition of the cell wall synthesis, intracellular translocation of AMPs, inhibition of protein/nucleic acid synthesis, and disruption of enzymatic/protein activity (20, 26). In both cases, electrostatic interaction is the key factor for the direct antimicrobial activity of cationic AMPs with the negatively charged molecules of the bacterial membrane, enabling further intrusion of the peptides into the inner part of the cell membrane (27). These interactions occur with the anionic phospholipids and phosphate groups of lipopolysaccharides (LPS) in case of Gram-negative bacteria as well with teichoic acids and lipoteichoic acids in case of Gram-positive bacteria (15, 24).

### Direct Antibacterial Activity

**Figure 2:**
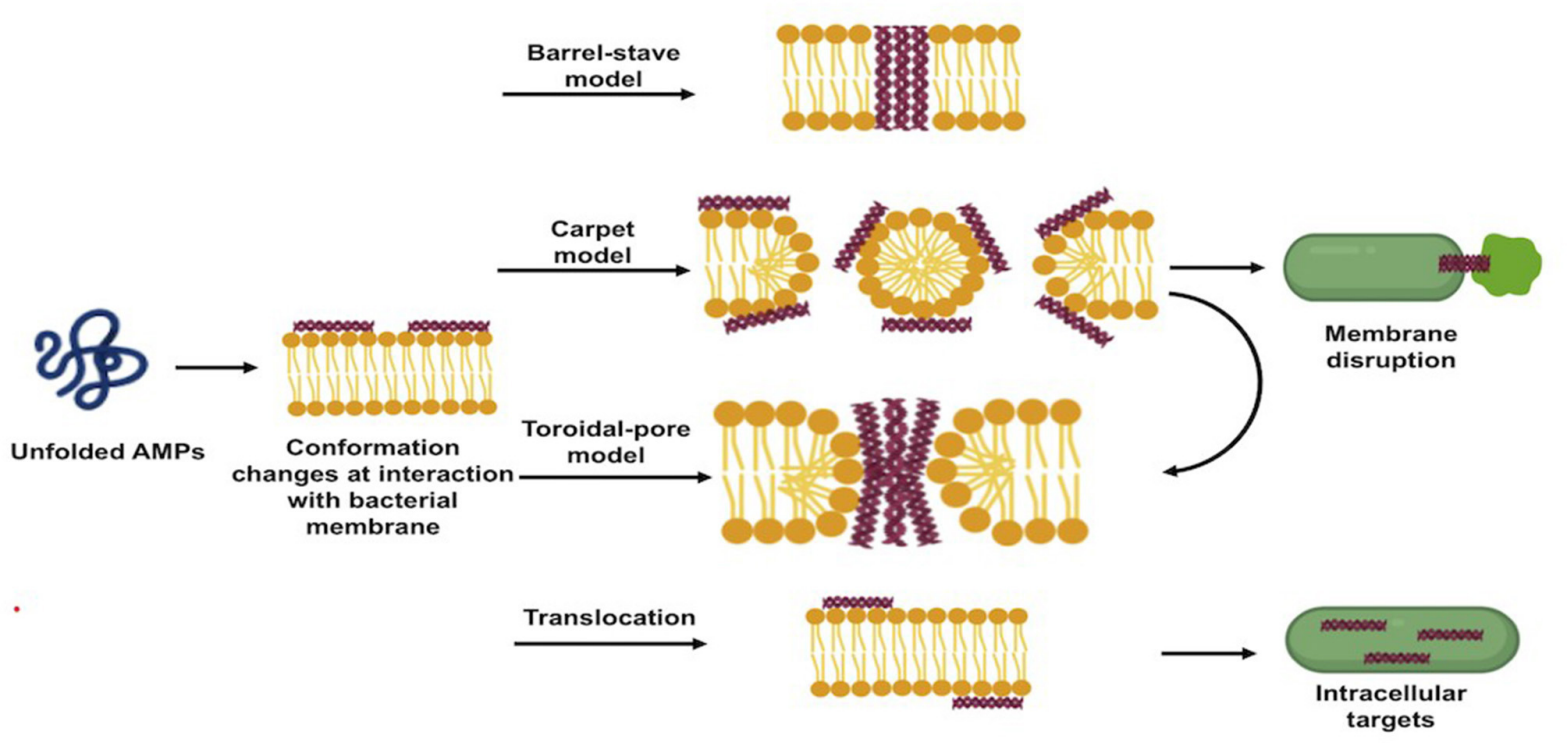
Schematic illustration of direct antibacterial mode of actions of antimicrobial peptides.

AMPs exert their direct antibacterial activity by either disrupting bacterial membranes or interfering with intracellular processes following to translocation. The direct antibacterial mechanism of AMPs is schematized in Figure 2.

#### Membrane Disruption Mechanism of Action

Upon adsorption into the membrane surface, the AMPs form, if not already present, an amphipathic secondary structure essential for interaction with the cell membrane (28). At this stage, several models have been proposed to describe the next events occurring at the bacterial cytoplasmic membrane, which ultimately lead to a remarkable dose-dependent membrane disruption (26). The three most popular models are the “barrel-stave pore model,” “toroidal-pore model,” and “carpet model” (10, 20).

In the barrel-stave pore model, when a threshold concentration of the peptides is reached, AMPs insert perpendicularly into the lipid bilayer forming transmembrane pores within the hydrophobic membrane core, in a manner similar to that of membrane protein ion channels. This model is consistent with the MOA of alamethicin, pardaxin, and protegrins (20, 26).

In the toroidal-pore model, once the minimum threshold concentration is reached, the peptides are perpendicularly incorporated into the bilayer membranes, enabling the lipid monolayers to curve around the pore. Consequently, the hydrophobic residues of peptides interact with the hydrophobic region of the membrane, forming pores that are partially bordered by the peptides and partially by the phospholipid head group, allowing the water core to be lined. Magainins and LL-37 adopt this MOA (10, 21).

In the carpet model, AMPs adsorb parallel to the lipid bilayer and cover the surface of the target membrane. Once their concentrations reach a certain threshold, AMPs exert detergent-like effects, which eventually disintegrate the membrane via the formation of micelles and pores. This model explains the MOA of cecropins and some magainins (10, 28). The formed pores act as non-selective channels for ions, toxins, and metabolites, thus preventing the microbe from maintaining vital homeostasis and leading eventually to microbial death (16, 27).

The carpet-like model is also called the “detergent-like model,” and the toroidal model is called the “wormhole model.” Table 2 shows different AMPs classified based on their membrane disruption mode of action.

**Table 2:** Classification of different antimicrobial peptides according to their membrane targeting mechanism of action.

**Pore-forming models**	**Example of AMP**	**Origin**
Barrel-Stave	Ceratotoxin Alamethicin Amphotricin B	*Ceratitis capitate* (Mediterranean fruit fly) *Trichoderma viride* (fungus) *Streptomyces nodosu* (bacteria)
Toroidal	Melittin LL-37 Piscidin Pardaxin	*Xenopus Laevis* (African clawed frog) *Homo sapiens* *Morone Saxtilis* (Atlantic striped bass) *Pardarchirus marmoratus* (Finless sole fish)
Carpet-like	Magainin 2 RL-37 Cecropins Dermaseptins Ovispirin Mastoparan X	*Xenopus Laevis* (African clawed frog) *Macaca mulatta* (Rhesus macaque) *Hyalophora cecropia* (North American moth) *Phyllomedusa spp*. (Frogs genus) *Ovis aries* (Sheep) *Vespa xanthoptera* (Japanese yellow hornet)

*AMP, antimicrobial peptide*.

#### Intracellular Targeting Mechanism of Action

Apart from the membrane-targeting MOA, some AMPs may exert other MOA, including the inhibition of extracellular wall synthesis and may have intracellular targets, thus disrupting intracellular processes (26). It has been shown that membrane permeabilization results in AMP translocation into the cytoplasm without disruption of its integrity, allowing binding to the anionic charge present in nucleic acids (DNA/RNA), some intracellular enzymes, and other targets (18, 26). For instance, AMPs, such as defensins, often confer antibacterial activity by interacting with various precursor molecules that are required for cell wall synthesis, such as the highly conserved lipid II (20). Other AMPs, such as indolicidin, interfere with protein synthesis, whereas papiliocin induces the production of oxygen free radicals, which damages both DNA and the cell membrane. Others can inhibit the activity of a few intracellular enzymes crucial for metabolism and proliferation of pathogens (26). Remarkably, it is suggested that AMPs may cause bacterial death via multiple and complementary actions known as a multi-hit mechanism, serving in increasing the efficiency of AMPs and evading resistance development (28).

### Immune Modulation

**Figure 3:**
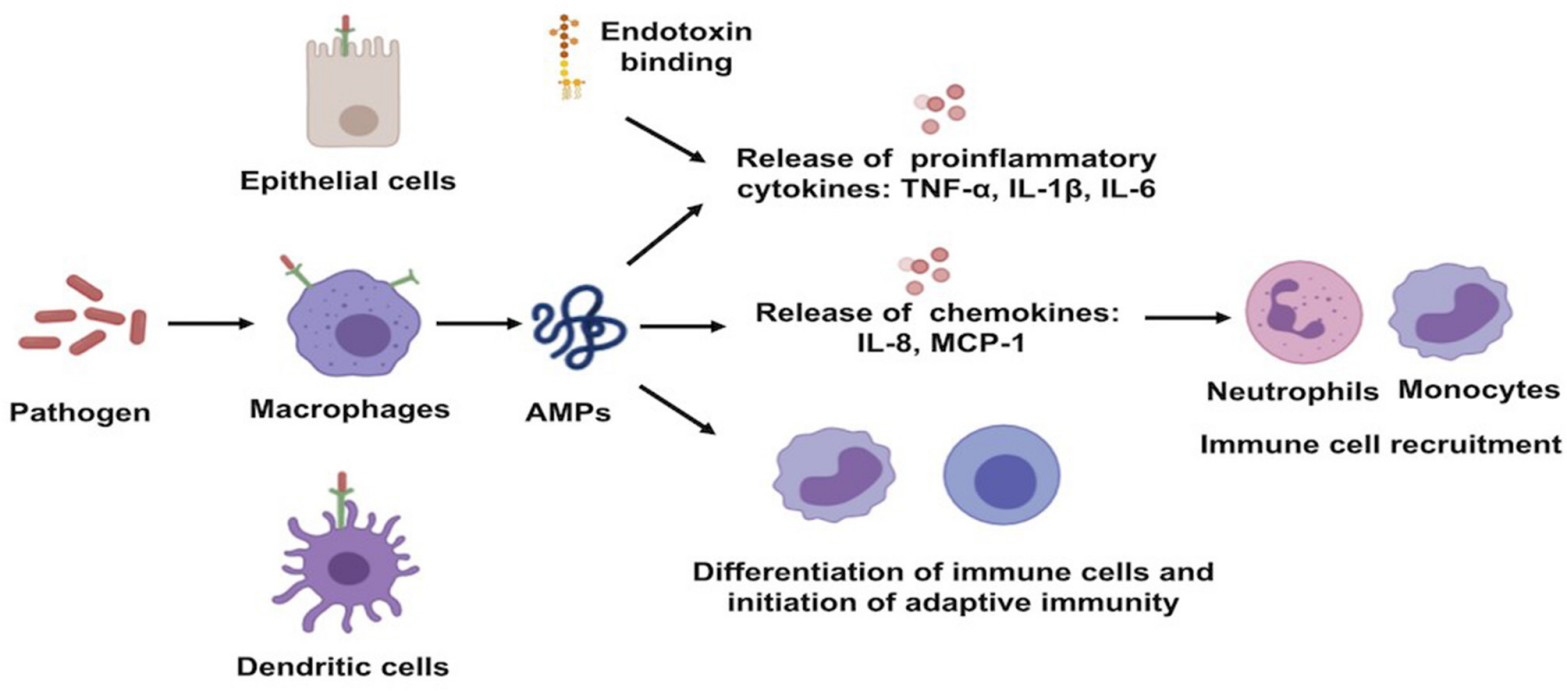
Schematic illustration of immune-regulatory functions of antimicrobial peptides. AMPs, antimicrobial peptides; IL, interleukin; MCP, monocyte chemoattractant protein; TNF-a, tumor necrosis factor alfa.

Well-characterized for their antimicrobial activities, AMPs are also known for their immuno-regulatory functions. The expression of these AMPs can be constitutive or can be inducible by infectious and/or inflammatory stimuli, such as proinflammatory cytokines, bacteria, or bacterial molecules that induce innate immunity (29). AMP production constitutes one of the early mechanisms by which the host immune system provides protection against invaders (13). They can recruit and activate immune cells, resulting in enhanced bactericidal activity and/or control of inflammation (20, 28). They act as effective inflammatory modulators by stimulating chemotaxis and angiogenesis, modulation of immune cell differentiation, and initiation of adaptive immunity. The broad range of mechanisms of action exerted by AMPs also includes toxin neutralization in an extremely rapid manner (Figure 3) (30). As examples, human neutrophil defensin (HNP)-1, HNP-2, and HNP-3 have been shown to upregulate the production of tumor necrosis factor alfa (TNF-α) and interleukin (IL)-1 by human monocyte activated upon bacterial infection, which, in turn, produces pro-inflammatory cytokines to attract immune cells to fight off the pathogens (31). In addition to that, HBD-2 and HBD-3 promote bacterial clearance of *Pseudomonas aeruginosa* by suppressing macrophage autophagy through downregulation of early growth response gene-1 (EGR1) and proto-oncogene c-FOS (32). Moreover, it has been demonstrated that cathelicidin exerts a direct chemoattractant action on monocytes, neutrophils, and T cells (33) and induces the transcription and release of IL-8 and monocyte chemoattractant protein (MCP)-1 and MCP-3, resulting in the recruitment of different immune cells requisite to remove the invading pathogen (34). LL-37, in addition to its direct MOA, neutralizes the activity of LPS and, thus, helps to protect the tissues from its harmful effects. In addition, it maintains a balance between pro- and anti-inflammatory mediators in the presence of LPS.

## Antimicrobial Spectrum of Activity

AMPs have broad-spectrum antibacterial activity and may exhibit their effects at minimum inhibitory concentrations (MICs) as low as 1–4 μg/ml (10). In addition to their potent antibacterial impact, some AMPs possess antiviral, antifungal, antiparasitic, and insecticidal properties. For instance, LL-37, the sole human cathelicidin, possesses a broad spectrum of activity against both Gram-positive and Gram-negative bacteria, such as *S. aureus, Enterococcus faecalis*, Group A *Streptococcus, Escherichia coli, P. aeruginosa, Klebsiella pneumoniae, Proteus mirabilis*, and *Prevotella intermedia* among others, including antibiotic-resistant strains containing methicillin-resistant *Staphylococcus aureus* (MRSA) and vancomycin-resistant *Enterococci* (VRE) (35–37). It has also a preventive action against *S. aureus* biofilm formation (38, 39) and can kill, *in vitro* and *in vivo*, both enveloped and non-enveloped viruses (40). Moreover, this peptide shows toxicity to tripomastigotes of the protozoan parasite *Trypanosama cruzi* at micromolar concentrations (35). On the other hand, magainins exhibit a broad spectrum of antimicrobial activity that includes Gram-positive and Gram-negative bacteria [*E. coli* (41) and *P. aeruginosa* (42)] and fungi, such as *Candida albicans* (43) at concentrations in the range of 1–10 μg/ml (44). The Type-IIA secreted phospholipase A_2_ (sPLA2-IIA) kills selectively Gram-positive bacteria (see below) while sPLA2-V contributes to the innate immune response against *C. albicans* by regulating phagocytosis and killing through a mechanism that is likely dependent on phagolysosome fusion (45). Defensins are also active against bacteria, fungi, and some viruses at low concentrations under optimal conditions (37). The antimicrobial activity of defensins is inhibited in the presence of increasing concentrations of salts and plasma proteins (44). Their spectrum of activity includes sexually transmitted infections causing pathogens, such as *Treponema pallidum, Chlamydia trachomatis*, human immunodeficiency virus (HIV)-1, and herpes simplex virus (HSV)-2 (43); fungal infections, such as candida species (43); skin infections due to *S. aureus* and *P. aeruginosa*; and other important bacterial pathogens, such as *Salmonella* and *Haemophilus influenzae* (46). Examples of peptides with their spectrum of activity are presented in Table 3.

**Table 3:** Example of peptides with their spectrum of activity.

**Targeted microbes/PAMP**	**Examples of AMP**
Gram-negative and –positive bacteria	IB-367, protegrin, MSI-78, gramicidin S, indolicidin, CEMA
Gram-negative bacteria Gram-positive bacteria	Polymyxin B HNP1, sPLA2-IIA
Fungi	Protegrin, indolicidin, gramicidin S, CEMA, polyphemusin, sPLA2-V
Virus	Indolicidin, protegrin, polyphemusin
Parasite	Magainin II, indolicidin
Endotoxin^*^	CEMA, polyphemusin variants

*AMP, Antimicrobial peptide; PAMP, Pathogen-associated molecular pattern*.

* *Endotoxin named also LPS, is a PAMP present in the cell wall of Gram-negative bacteria*.

AMPs are generally capable of killing microbes independently. However, they often show enhanced antimicrobial activity when tested in combination with either other AMPs or conventional antibiotics (7, 35). Many previous studies have shown that the use of antibacterial agents in a therapeutic cocktail can reduce the dose of each drug in the combination, limiting the development of resistance *in vitro* (18). For instance, LL-37 and HNP-1 were shown to work synergistically together with a significant enhancement of both their antimicrobial activities and membrane permeabilization effects (35). It has been also demonstrated that the efficacy of conventional antibiotics could be further boosted through combination with AMPs, and some studies revealed synergistic relationships between antibiotics and AMPs (47, 48). For example, Dosler and Mataraci reported the synergistic effect of indolicidin combined to conventional antibiotics daptomycin, teicoplanin, and ciprofloxacin against MRSA biofilm (48). Furthermore, our recent studies showed that the AMP LL-37 potentiated the bactericidal effects of the antibiotics colistin and imipenem on both antibiotic susceptible and multidrug resistant strains of *P. aeruginosa* (49).

## Type-IIA Secreted Phospholipase A_2_: A Particular Host Antimicrobial Peptide

The type-IIA secreted phospholipase A_2_ (sPLA2-IIA) is a member of the super-family of enzymes called sPLA2 originally defined by their ability to catalyze the hydrolysis of phospholipids from both eukaryotic and prokaryotic cell membranes at the *sn*-2 position leading to the generation of lysophospholipids and free fatty acids (50, 51). The sPLA2-IIA can be classified as a member of the AMP family although it kills bacteria via a different MOA (see below) and is larger than most AMPs (120 amino acids). The classifications of sPLA_2_ in different types is based on the number and position of their disulfide bridges (50, 51). The encoding sequences of some sPLA_2_ complimentary DNA (cDNA) predicted the presence of the putative signal peptide, thus indicating that these types of sPLA_2_ are secreted proteins. To date, 10 distinct members of sPLA_2_s have been identified so far in mammals with around 50% homology among them (50, 51). It becomes clear now that sPLA2-IIA is a major actor in host defense against invading bacteria and is produced by host cells at sufficient levels to ensure this role (52, 53).

### Discovery of the Bactericidal Functions of sPLA2-IIA

sPLA_2_-IIA, the most studied enzyme of the sPLA2 group, is the most abundant in human and animal biological fluids, and it has been initially proposed to play a role in the pathogenesis of various inflammatory diseases (50, 51). However, this notion evolved progressively, and it is now accepted that bacterial killing represents the most physiologically relevant and recognized function of sPLA2-IIA (52, 53). The group of J. Weiss reported for the first time that the potent antistaphylococcal activity present in the inflammatory peritoneal exudate can be attributed mostly to sPLA2-IIA (54). This bactericidal effect is due to the ability of sPLA_2_-IIA to bind and penetrate the cell wall of Gram-positive bacteria with greater efficiency compared to its Gram-negative effect (52, 53, 55). Subsequent studies report that mouse and human sPLA_2_ exhibit various bactericidal activities toward two Gram-positive bacteria, *Listeria monocytogenes* and *S. aureus*, and that sPLA_2_-IIA is, by far, the most bactericidal sPLA_2._ The concentrations of sPLA_2_-IIA in biological fluids are sufficient to kill all Gram-positive bacteria that may infect mammals (52, 53). Whereas, the concentrations of sPLA_2_-IIA in the normal human tear exceed 30 μg/ml, only 1.1 ng/ml of the enzyme is sufficient to achieve the killing of *L. monocytogenes* (56). Concentrations at 15–80 ng/ml of sPLA2-IIA are necessary for *S. aureus* killing. The sPLA_2_-IIA efficiently kills Gram-positive bacteria due to the high net positive charge of this enzyme compared to that of other sPLA_2_s, allowing rapid and highly efficient binding of sPLA_2_-IIA to the negatively charged surface of these bacteria (52, 53). The cell wall bacterial component lipoteichoic acid has been reported to play a key role in the tight binding of sPLA2-IIA to Gram-positive bacteria, such as *S. aureus* (57).

The contribution of sPLA2-IIA to antibacterial host defense is supported by *in vivo* experiments using sPLA2-IIA Tg mice (52, 53). sPLA2-IIA Tg mice were generated in the C57Bl/6 background. This mouse strain contains an inactivating point mutation in murine sPLA2-IIA, making them natural knockouts (58, 59). Therefore, expression of sPLA2-IIA in this strain background is not confounded by the co-expression of murine sPLA2-IIA. Using these mice, it has been established that sPLA2-IIA protects from lethal infections of *S. aureus, Bacillus anthracis*, and *Streptococcus pyogenes* (60–64).

## Amps and Diseases

The skin or the mucosal surface, such as the respiratory tract, the gastrointestinal tract, and the urogenital tract (11), are classically considered as the primary sites at which a host encounters a pathogen. At these sites, infections are controlled by the innate defense responses that allow the host to maintain its integrity (12). Knockout mice and Tg expression systems have confirmed that AMPs play a central role in limiting microbial proliferation in various host sites, thus preventing spread to the deep tissues where serious infection may occur (14). AMPs are produced by epithelial cells of vertebrates as a first line of defense against microbial invaders and tend to exhibit intrinsic specificity for the encountered pathogens. For instance, HNPs are expressed at high levels in lesions of superficial folliculitis due to skin infection by *S. aureus* (11). As an initial part of the inflammatory response, AMPs are produced by inflammatory cells, such as neutrophils and tissue phagocytes, including macrophages (31). For example, HBD is upregulated in monocytes exposed to bacteria, LPS, or IFNɤ (65). Furthermore, the immunomodulatory activities of AMPs enable the activation of adaptive immune responses. LL-37 represents a classical example of AMPs that binds to LPS leading to repressed LPS-induced responses and targeting the NF-κB pathway. Moreover, studies have shown that a downregulation of AMP expression is associated to an increase in susceptibility to infections by viruses and other microorganisms (13).

AMPs play an integral role in a large number of respiratory diseases [for example, tuberculosis, cystic fibrosis (CF), rhinitis, etc.], gastrointestinal diseases (shigellosis, inflammatory bowel disease, etc.), and cutaneous diseases (atopic dermatitis, psoriasis, wound healing, and rosacea) among others (13, 66). Group B Streptococcus (GBS) is killed by human serum from patients with GBS-related infections in an sPLA2-IIA-mediated manner (63). In healthy patients, sPLA2-IIA is the only sPLA2 isoform that is constitutively present at low ng/ml concentrations in the circulation (67–69). Increased levels of sPLA2-IIA have been observed in biological fluids in various inflammatory and infectious diseases, such as allergic rhinitis, rheumatoid arthritis, pancreatitis, septic shock, acute respiratory distress syndrome (ARDS), or CF, and correlated to symptom severity of these diseases (50, 70). However, it remains unclear whether upregulation of sPLA2-IIA expression is the cause and/or the consequence of inflammation (e.g., increased cytokine production) in these diseases. Elevated sPLA2-IIA levels have also been observed in arterial plasma and in bronchoalveolar lavage fluids of patients with septic shock. These levels have a prognostic value and correlated with the development of pulmonary failure (50). We focus in more detail in the following paragraph, the potential relevance of AMPs in CF.

### Cystic Fibrosis

Patients with disruptions in lung immunity or mucosal clearance, such as patients with CF, suffer from bacterial infections that typically don't resolve even with antibiotic treatment (71). CF is a well-characterized, lethal, autosomal, recessive, inherited disorder found predominantly in Caucasians due to mutation in the cystic fibrosis transmembrane conductance regulator (CFTR) gene, characterized by chronic lung bacterial infections (72). These infections are major causes of morbidity and mortality of CF patients. Ultimately, 80 to 95% of patients with CF succumb to respiratory failure brought on by these chronic bacterial infections associated with airway inflammation (73). *P. aeruginosa* is arguably the major colonizing infection for people with CF (74).

The main AMPs detected in lung tissues and secretions of CF patients are neutrophil α-defensins/HNPs, HBDs, LL-37, and sPLA2-IIA that play a major role in lung immunity and protect them against infection with harmful microorganisms (75). The persistence of lung bacterial infection may be partly explained by an acidification of the airway surface liquid (ASL) within the CF lung that exhibits reduced bacterial killing due to the compromised function of AMPs (72, 76). Our recent studies showed that ASL was significantly more acidic in CF than in wild-type (WT) respiratory cells. This was consistent with a defect in bicarbonate secretion involving CFTR and SLC26A4 (pendrin) and a persistent proton secretion by ATP12A. This was associated to a defect in *S. aureus* clearance, which was improved by pH normalization (72).

Abnormal salinity of ASL has also been suggested to impair the bactericidal activity of AMPs, which can form bacterial proliferation within CF airways (77). We recently showed that the defensin BigDef1 from the oyster *Crassostrea gigas* exhibits natural salt-stable and broad-range bactericidal activity against various bacterial species. We took advantage of this salt-stability, due to an evolutionary adaptation of oyster defensins to sea environment, to treat bacteria from CF patients. We showed that BigDef1 efficiently kills multidrug-resistant clinical isolates of *S. aureus* from CF patients even at high salt concentrations (78).

In the early stages of CF, the airways are mainly colonized by *S. aureus*, whereas in later stages, *P. aeruginosa* is the major pathogen (46). This shift in infection is a characteristic feature of CF. Once it colonizes the CF airways, *P. aeruginosa* induces a robust expression and secretion of sPLA2-IIA by airways epithelial cells via a Krüppel-like transcription factor (KLF)-2-dependent pathway, leading to subsequent and selective killing of *S. aureus* by sPLA2-IIA, a process contributing to the infection shift (16). A similar phenomenon has been reported during periodontal diseases caused by *Porphyromonas gingivalis*. The latter induces sPLA2-IIA production and secretion by oral epithelial cells via activation of the Notch-1 receptor (45). The sPLA2-IIA concentrations reach levels leading to the killing of other oral bacteria much more susceptible to this enzyme sPLA2-IIA compared to *P. gingivalis* (45). This process is a potential cause of dysbiosis associated with periodontal disease. Thus, it is of great importance to examine the role of individual bacterial species within the microbiome in the induction or inhibition of sPLA2-IIA expression at mucosal sites and whether this may contribute to occurrence of dysbiosis at mucosal surfaces in diseases characterized by polymicrobial infections.

## Advantages of Amps and Challenges

As the emergence of super-bacteria is causing a serious concern across the globe, researchers are working on the development of new anti-infective therapies. Among the alternatives to combat antimicrobial resistance, AMPs have garnered much attention over the years (79). AMPs, which are widely expressed in all kind of living organisms and have been preserved in the long evolutionary process, are with no doubt effective natural immunologically active molecules (80). AMPs have excellent *in vitro* antimicrobial activity against a wide range of microbes and, therefore, represent a promising alternative to combat resistance (18). The rapid bactericidal activity of AMPs constitutes a strong advantage to the future of peptide-based antibacterial therapy. In addition, AMPs are active against multidrug-resistant bacteria (49, 81). Furthermore, AMPs possess concomitant broad anti-inflammatory and immunomodulatory activities. Besides, AMPs exhibit synergistic or additive effects upon co-administration with conventional ATBs to treat both susceptible and multidrug-resistant bacteria at non-toxic concentrations (70, 71).

Due to the overlapping MOA of AMPs involving multiple low-affinity targets, unlike the MOA of conventional ATBs characterized by one defined, high-affinity target, the development of bacterial resistance toward AMPs has generally been considered to be improbable (28, 82). In particular, given that the bacterial cell membrane is the primary target of AMPs, it is challenging for microbes to preserve the cell membrane functional and structural integrity while at the same time avoiding the membrane disruption activity of AMPs (28). Because the AMP is composed of amino acids with no specific primary sequence signature, the microbe is unable to synthesize a protease that can cleave the AMP but not its own proteins. Furthermore, our recent study showed that the AMPs LL-37 and CAMA, a derivative of cecropin, were associated with only transient and low levels of induced resistance compared to the induced resistance by the antibiotic gentamicin (49). However, it appears somehow that some bacteria, such as *Serratia marcescens*, present natural resistance to AMPs (83). Moreover, some bacteria exposed to AMPs may evolve under selective pressures to develop resistance mechanisms. Even though the existence of these selective pressures are, evolutionarily speaking, quite old, human AMPs still possess a broad spectrum of effective activity against a diverse range of microorganisms (14).

In the last 30 years, various pharmaceutical companies have tried to develop AMPs as clinically useful antimicrobials. To date, several AMPs are currently undergoing laboratory testing, and a few have already reached clinical trials (19). The review (18) shows a number of AMPs and AMP derivates already at the preclinical stage and in clinical trial.

Although AMPs have very attractive qualities, the challenges for successful development for clinical application are considerable (84). One of the biggest restraints in the large scale of development and commercialization of AMPs may be their high production costs estimated around US$300–$500 per gram, which is several hundred times more expensive than the production of conventional ATBs (17). In addition to that, the excellent antimicrobial activity *in vitro* is rarely translated *in vivo* (41). In most studies in the field, the killing effects of AMPs on bacteria have been examined *in vitro* and in the absence of host cells, which do not reflect real life. Indeed, in human and animal infectious diseases, infecting bacteria multiply within biological fluids and/or in contact with host cells, which may interfere with AMP bactericidal activity. This led us to compare the bactericidal effects of LL-37 on *P. aeruginosa* in a cell-free system and when this strain was added to a bronchial epithelial cell line IB3, isolated from a CF patient, prior to addition of LL-37. These studies show that the presence of IB3 cells markedly reduces the bactericidal effects of LL-37 on *P. aeruginosa*. Although the mechanisms involved in this alteration are still under investigation, we hypothesized that degradation of LL-37 by a protease produced by IB3 cells upon infection by *P. aeruginosa* may explain the alteration of LL-37 bactericidal activity (unpublished data). Thus, most peptides have relatively short circulating plasma half-lives and are cleared primarily by proteolytic degradation and by renal filtration, generally leading to suboptimal pharmacokinetic properties (84). Indeed, the most obvious cause of poor or incomplete *in vivo* activity of AMPs is the lack of stability due to the peptide susceptibility to protease degradation if they are ingested. In regard to drug delivery, oral bioavailability of peptides is often no more than 2% (79). Thus, oral administration of AMPs can lead to proteolytic digestion by enzymes in the digestive tract, such as trypsin and pepsin, making intravenous or subcutaneous injections the only viable routes of administration to treat people. Moreover, systemic administration outcomes with short-half lives *in vivo*, protease degradation, and cytotoxic profiles in blood (20). In addition, the direct antibacterial activity of some of these AMPs is certainly prevented due to the affinity of these AMPs to polyvalent anions, such as glycosaminoglycans (29). AMPs can also bind avidly to host cells, which may reduce their availability to bind to and kill bacteria (unpublished data).

Another key factor to consider is the potential of these peptides to elicit an immunogenic response that can significantly reduce their efficacy and alter their pharmacokinetic profile (84). An additional challenge to overcome is the differences in pH, salt, and serum concentrations *in vivo*, resulting in decreased antimicrobial activity (10). Hence, a number of AMPs have failed approval by the FDA after reaching phase II clinical trials due to their short-half life and their poor physical-chemical properties (17). However, even with limitations, AMPs still possess a broad spectrum of potent antimicrobial activity (14). Another potential issue includes the cytotoxicity to mammalian cells when bactericidal concentrations are high. However, there are very few studies of AMP cytotoxicity on human cells (10).

Methods to overcome these challenges have been evaluated. Scientists and pharmaceutical companies have invested in research and development to overcome the barriers limiting the practical application of AMPs. To circumvent proteolysis, sequence modifications, and half-life, advances in peptide formulation have ended in the development of improved formulas with sufficient plasma exposure using a delivery system (for example, a lipid self-assembly system, inorganic systems, nanoparticles, etc.), chemical modifications of AMPs, and altering structure to have cyclic peptides with strained peptide bonds displaying a resistant profile (41, 85). Another approach is to identify possible molecular cleavage sites followed by substitution of the relevant amino acids (86). The recognized route of administration for therapeutic peptides remains parenteral, in which AMPs pierce the membrane barriers where they are poorly absorbed. Nevertheless, other challenges remain, pre- and post-administration, in achieving both the desired pharmacokinetic profile and high patient compliance (84). Specific cell-penetrating peptide sequences have been identified and can be used to transport AMPs across membranes (79). More tools to increase AMP activity include modifications in charge and hydrophobicity. Among the various methods for peptide optimization, quantitative structure-activity relationship, and the introduction of fluorine atoms or trifluromethyl groups have been recently used (25). Besides, recent studies have focused on designing a sequence of AMP analogs with modified yet improved antibacterial, cytotoxic, and hemolytic activities. Thus, synthetic peptides have been designed to mimic the structure, function, and mode of action of AMPs with enhanced properties, resulting in low cytotoxicity and high resistance to proteolytic degradation, resulting in prolonged half-lives and cost-effective molecules (18). Furthermore, the progress in designing non-immunogenic peptides is rapid, resulting in disarming the immunogenic response, which should increase clinical success (84).

## Conclusions and Perspectives

AMPs, owing to their broad spectrum of antibacterial activity and their effectiveness against multidrug-resistant bacteria, are a promising replacement for conventional ATBs, invoking a multi-hit mechanism that cannot be easily overcome by bacteria. However, the future of peptide-based anti-infective drugs is still uncertain. The major barriers that hinder their clinical use are mainly their stability *in vivo*, their non-well-studied toxicity, and their high production costs. Thus, the development of optimal formulations of AMPs at a reasonable cost, finding the preferred route of their administration, and evaluating their cytotoxicity remain the main interest to scientists. Regardless of the field of applications, AMPs constitute the most promising drug candidate in a foreseeable future in overcoming the alarming rise of bacterial resistance.

## Author Contributions

RG and CM wrote the article. ZX did the figures and tables. LT and DK revised and corrected the manuscript. All authors read and approved the final manuscript. All authors contributed to the article and approved the submitted version.

## Conflict of Interest

The authors declare that the research was conducted in the absence of any commercial or financial relationships that could be construed as a potential conflict of interest.

## Abbreviations

AMPs: Antimicrobial peptides
ARDS: Acute respiratory distress syndrome
ASL: Airways surface liquid
ATBs: Antibiotics
cDNA: complimentary DNA
CF: Cystic fibrosis
CFTR: cystic fibrosis transmembrane conductance regulator
EGR1: Early growth response gene-1
GBS: Group B Streptococcus
HBD: Human β-defensin
HIV: Human immunodeficiency virus
HNP: Human neutrophil defensin
HSV: Herpes simplex virus
IL: Interleukin
KLF: Krüppel-like transcription factor
LPS: Lipopolysaccharides
MCP: Monocyte chemoattractant protein
MIC: Minimum Inhibitory Concentration
MOA: Mechanism of action
MRSA: methicillin-resistant *Staphylococcus aureus*
PAMP: Pathogen-associated molecular pattern
sPLA2: secreted phospholipase A2
sPLA2-IIA, Type-IIA secreted phospholipase A_2;_TNF-α: Tumor necrosis factor alfa
Tg: Transgenic
VRE: vancomycin-resistant *Enterococci*
WT: Wild-type.
